# Development and Validation of the Scan of Postgraduate Educational Environment Domains (SPEED): A Brief Instrument to Assess the Educational Environment in Postgraduate Medical Education

**DOI:** 10.1371/journal.pone.0137872

**Published:** 2015-09-28

**Authors:** Johanna Schönrock-Adema, Maartje Visscher, A. N. Janet Raat, Paul L. P. Brand

**Affiliations:** 1 Center for Educational Development and Research in health sciences, Institute for Medical Education, University of Groningen and University Medical Center Groningen, Groningen, The Netherlands; 2 UMCG Postgraduate School of Medicine, University of Groningen and University Medical Center Groningen, Groningen, The Netherlands; 3 Princess Amalia Children’s Centre, Isala Hospital, Zwolle, The Netherlands; University of Michigan, UNITED STATES

## Abstract

**Introduction:**

Current instruments to evaluate the postgraduate medical educational environment lack theoretical frameworks and are relatively long, which may reduce response rates. We aimed to develop and validate a brief instrument that, based on a solid theoretical framework for educational environments, solicits resident feedback to screen the postgraduate medical educational environment quality.

**Methods:**

Stepwise, we developed a screening instrument, using existing instruments to assess educational environment quality and adopting a theoretical framework that defines three educational environment domains: content, atmosphere and organization. First, items from relevant existing instruments were collected and, after deleting duplicates and items not specifically addressing educational environment, grouped into the three domains. In a Delphi procedure, the item list was reduced to a set of items considered most important and comprehensively covering the three domains. These items were triangulated against the results of semi-structured interviews with 26 residents from three teaching hospitals to achieve face validity. This draft version of the Scan of Postgraduate Educational Environment Domains (SPEED) was administered to residents in a general and university hospital and further reduced and validated based on the data collected.

**Results:**

Two hundred twenty-three residents completed the 43-item draft SPEED. We used half of the dataset for item reduction, and the other half for validating the resulting SPEED (15 items, 5 per domain). Internal consistencies were high. Correlations between domain scores in the draft and brief versions of SPEED were high (>0.85) and highly significant (p<0.001). Domain score variance of the draft instrument was explained for ≥80% by the items representing the domains in the final SPEED.

**Conclusions:**

The SPEED comprehensively covers the three educational environment domains defined in the theoretical framework. Because of its validity and brevity, the SPEED is promising as useful and easily applicable tool to regularly screen educational environment quality in postgraduate medical education.

## Introduction

In postgraduate medical education (PGME), the quality of the educational environment is important for the effectiveness of clinical teaching programs [1,2], for trainee satisfaction [3], and for wellbeing and quality of life [4]. Although various instruments have been developed to evaluate the quality of medical educational environments [2,5,6], these instruments measure different concepts, which raised the thought that existing instruments might lack a common solid and universally accepted theoretical basis. A recent study substantiated this thought [3]. Further investigation [3] led to the identification of a solid general theoretical framework [7,8], which defines three key domains describing human environments. This general framework has been validated in different contexts [7–9], including the educational context, and appeared to be also applicable to the more specific medical education environment [3]. We aimed to develop an instrument–based on this theoretically sound framework and suitable for regular use–that solicits feedback from residents to screen the quality of the educational environment in postgraduate medical education (PGME) programs.

The central idea behind the general theoretical framework for describing human environments is that each kind of human environment can be characterized in terms of three common sets of dimensions, or in other words: in terms of three broad domains [7–9]. Research provided support for this theory [7–9]: apart from some slight variations, similar kinds of dimensions were found in different kinds of human environments like psychiatric wards, correctional institutions, psychiatric treatment programs, military training settings, university student residences, high school classrooms and work environments, namely *personal development or goal direction dimensions*, *relationship dimensions* and *system maintenance and system change dimensions*.

The personal development or goal direction domain concerns the basic goals of the environment and comprises the aspects in the environment that are important for personal growth and self-enhancement. For instance, the main aim of educational settings is achieving the learning goals. To achieve these goals, it is important that the educational environment scores high on aspects like clarity about learning objectives, relevance of learning content and providing constructive criticism.

The relationship domain concerns the involvement of people in the setting, the extent to which they support each other and to which they feel free to express themselves in an open and spontaneous way. A beneficial relationship domain implies that there is open communication, that people in the setting are friendly toward each other and that they provide social and interpersonal support to each other. In addition, they have a clear sense of cohesion and of group spirit. In educational settings, dimensions relevant to assess positive relationship are, for instance, student involvement, (emotional) support, affiliation or bonding, and support by teachers.

The system maintenance and system change domain concerns the degree to which there is order in the environment and clarity in expectations, the amount of control in the environment and the environment’s responsiveness to change. In educational settings, typical dimensions to assess this domain are organization, order, rule clarity, teacher control, student influence and innovation. In specific learning environments like workplace-learning settings, some dimensions relevant to work environments may also apply, for instance, work pressure and physical comfort.

A content analysis of existing instruments to assess the medical educational environment showed that the majority of the items in these instruments could be related to the general framework. In the medical education context, the contents of the three domains could be summarized as *goal orientation*, *relationships* and *organization/regulation* [3]. Goal orientation relates to the content of the program; the relationships domain covers the interpersonal aspects and atmosphere of the program; and organization/regulation comprises the structure and organization of the program.

We aimed to contribute to the medical education environment field by applying a solid theoretical framework–i.e. a theoretical framework that has been tested repeatedly and that appears to be generally acknowledged [10]. In literature, a strong conceptual or theoretical framework and building on prior work have been emphasized as vital for the quality of scholarly endeavours [11–13]. Several existing instruments to assess the medical educational environment used literature [14,15] or previous instruments [16–20] as a basis for their development processes. Some went a step further as they used the outcomes of a qualitative study or a systematic review as a conceptual framework for the development of their instruments [19,20]. We aimed to extend these previous efforts by integrating prior work with an established solid theoretical framework that was tailored to the medical education setting [3,7–9].

The purpose of this study was to develop a theoretically sound instrument–suitable for regular use–that solicits resident feedback to screen the quality of the educational environment in postgraduate medical education (PGME) programs. We based the instrument on the aforementioned theoretical framework defining goal orientation, relationships and organization/regulation as the three key domains of the medical educational environment. In addition, we built on prior research by using the contents of previously developed environment instruments and the outcomes of the content analysis described earlier [3]. As a result, the new instrument presented in this paper reflects the integration of a solid theory and the content of existing instruments to assess PGME environment quality.

Considering our objective to develop an instrument that is suitable for regular use, we aimed to limit its length. Existing instruments assessing the quality of the educational environment in PGME [2,3,6] are relatively long, with up to 50 items [19,20]. This may not be a major problem during the development and validation phase of an instrument, when developers can strive for high response rates, even for comprehensive instruments. In general, however, response rates are inversely related to questionnaire length [21,22]. Particularly when respondents are regularly asked to complete evaluation instruments, questionnaire length may cause evaluation fatigue and reduce response rates [23]. A brief instrument with a limited number of items is therefore preferable. In summary, the aim of the present study was to develop a valid, theoretically sound and yet concise instrument to assess the quality of the PGME environment on a regular basis: the Scan of Postgraduate Educational Environment Domains (SPEED).

## Methods

### Development of the SPEED

We developed the SPEED in a stepwise fashion. First, we extracted items from published instruments assessing the educational environment. Building on previous work in which we categorized all the items from eight existing instruments into the three domains goal orientation (content), relationships (atmosphere) and organization/regulation (organization) [3], we added items from two additional instruments published after completion of that review [20,24] using the same procedure. We judged the items on their relevance and applicability to assess the educational environment quality in postgraduate medical education. If necessary, we tailored items in such a way that they applied to only one domain. We excluded items that did not address the educational environment and items specifically aimed at one discipline or specific curriculum, and we merged duplicate items. Subsequently, we used a modified Delphi procedure to reduce the number of items in the instrument [25,26]. We asked eight physicians and educationalists with ample experience in studying the educational environment in PGME to choose ten items each from the item list of each domain. They were instructed to choose the items that they considered most important, and to ensure that the chosen items comprehensively covered all three educational environment domains (content, atmosphere and organization), and then to rank these items by order of relevance. Items that were selected by at least three of the eight experts were included in an initial draft version of the SPEED. This initial draft was presented to the eight experts, along with a list for each domain of additional items that had either been identified by two experts instead of three, or were considered by one expert as one of the three most relevant items. In this second Delphi round, the eight experts were asked to select no more than three items per domain from this additional list of items, and once again choose those items they considered most important and required for full coverage of the domain in question. Items that were selected by three or more experts in this stage were added to the draft instrument. The resulting second draft of the SPEED was compared to results of semi-structured interviews with 26 residents in three teaching hospitals (10 in a general teaching hospital and 16 in two university teaching hospitals). In these interviews, residents were asked to identify issues that they thought had the greatest impact on the quality of their PGME training program. Issues that were mentioned by at least 4 residents and that had not been covered by items in the draft version of the SPEED were added to the instrument item list by selecting–through investigator triangulation–items from the initial item bank that addressed these issues appropriately. The instrument was circulated among the eight experts for final additions, deletions, or comments. The result was the final draft version of the SPEED.

### Item reduction and validation

The final draft of the SPEED was sent as a web-based survey to all PGME residents in a general hospital (Isala Hospital, Zwolle) and in a university hospital (University Medical Centre Groningen) in the Netherlands. Respondents were asked to score their agreement to each item (presented as a statement) on a 4-point Likert scale ranging from 0 (completely disagree) to 3 (completely agree). In addition, respondents were asked to provide an overall rating of the quality of their PGME training program in the three domains (content, atmosphere, and organization) as a numerical score ranging from 1 (very poor quality) to 10 (excellent quality). This method of providing an overall rating of performance on a scale ranging from 1 to 10 is the standard way of grading tests and exams in schools and universities in the Netherlands.

Item reduction was planned in two stages. First, we calculated correlation coefficients between the mean item score in each domain and the overall numerical score for that domain. If the correlation coefficient between the mean item score and the total numerical score was >0.85, the domain in the abbreviated instrument would be reduced to only the overall numerical score. If this correlation was ≤0.85, item reduction was performed on half of the database (the derivation dataset), and validation of the abbreviated instrument on the other half (the validation dataset). As item reduction based on only maximization of internal consistencies may bear the risk of reduced validity due to redundancy in the items and limited coverage of each of the three domains, we followed published recommendations to not limit item reduction to internal consistency alone, but also take item content into account [27]. Therefore, we clustered the items within each domain into themes. We aimed to select at least one item within each subject theme for inclusion in the final, abbreviated version of the SPEED to ensure full coverage of each domain, and we aimed to optimize internal consistencies by taking item-total correlations into account [27]. We considered items eligible for inclusion in the abbreviated SPEED if the adjusted item to total domain score correlations were at least 0.40 in the derivation dataset, and not below 0.30 in the validation dataset. We intended to develop an abbreviated instrument of 15 items (no more than 5 in each domain).

Using the validation dataset, we validated the abbreviated SPEED by calculating the adjusted item-to-total-domain-score correlations of each item (required not to be <0.30) [28], the correlations between domain scores of the final draft and abbreviated versions of the instrument, and the degree of variance in the final draft domain scores explained by the abbreviated domain scores using multiple linear regression models.

### Ethics

The study was conducted according to the declaration of Helsinki, and was approved by the ethical review board of the Dutch Society of Medical Education (NERB dossier number 171). All participants provided informed consent.

## Results

### Development

After item extraction and deletion of duplicate items, the first draft of the SPEED consisted of 169 items: 67 in the content domain, 48 in the atmosphere domain, and 54 in the organization domain. After the first Delphi round, these were reduced to 10, 11, and 10 items, respectively. In round 2, 7 items were added (3, 1 and 3, respectively). After the interviews with residents, 5 more items were added: 4 to the content and 1 to the organization domain. The final draft SPEED therefore consisted of 43 items (see Table 1). There were 5 themes in the content and atmosphere domains, and 3 in the organization domain.

**Table 1:** Items in the final draft version of the Scan of Postgraduate Educational Environment Domains (SPEED), grouped per domain and subject theme, with their adjusted correlation coefficients to total domain score in the derivation dataset^a^.

domain	subject theme		Item-to-total correlation
**Content**	Purposiveness	**The training in this post prepares me for my future career**	0.65
(Cronbach’s α 0.88)		The clinical learning opportunities in this program include all required clinical skills	0.48
		This post includes all adequate practical and clinical skills to be acquired	0.47
		The formal educational program is targeted to my learning needs	0.44
	Teaching style	**My supervisors are all in their own way positive role models**	0.56
		I am asked on a regular basis to provide a rationale for the clinical choices I make and for my actions	0.48
		Senior staff utilize learning opportunities effectively	0.44
		Handover is being used as a teaching opportunity	0.15
	Appraisal and feedback	**The feedback provided by my supervisor is focused on my strengths and weaknesses**	0.65
		My supervisor bases feedback on concrete observations of my performance	0.62
		My supervisor not only appraises my medical expertise but also other competencies such as teamwork, organization or professional behaviour	0.57
	Independence and	**The level of autonomy given to me is appropriate to my level of training**	0.55
	responsibility	The level of clinical responsibility given to me is appropriate to my level of training	0.55
		My supervisor gives me enough freedom to independently perform tasks that suit my current knowledge and skills levels	0.54
		My supervisor encourages me to find out things for myself	0.48
	Monitoring progress	**In this rotation, evaluations are useful discussions about my performance**	0.63
		In assessment interviews, the program director provides a clear link to previously defined learning goals	0.55
**Atmosphere**	Respect	**The supervisors are respectful towards residents**	0.75
(Cronbach’s α 0.90)		There is an atmosphere of cooperation and mutual respect in this department	0.72
		The supervisors promote an atmosphere of mutual respect	0.70
		Handover takes place in a safe climate	0.65
	Team spirit	**Supervisors, nursing staff, other allied health professionals and residents work together as a team here**	0.71
		I feel part of the team working here	0.67
	Relations and atmosphere	**There is (are) NO attending physician(s) who have a negative impact on the educational climate**	0.59
		I have a good sense of rapport with senior staff in the department	0.56
	Accessibility	**The supervisors are approachable and helpful**	0.59
		When I need to consult a supervisor, s/he is readily available	0.49
	Support	**My supervisor supports me in difficult situations (e.g. handover)**	0.59
		My program director helps and advises me on how to create and/or maintain a good work-home balance	0.43
**Organization**	Organization	**Teaching and learning are emphasized in this department**	0.43
(Cronbach’s α 0.82)	attuned to learning aims	I am given relief from clinical duties to be able to participate in the formal educational programme	0.43
		Residents are generally able to attend scheduled educational activities	0.38
	Organization	**My program director reserves time to supervise/counsel me**	0.60
	of supervision	**Good clinical supervision is available at all times**	0.54
		Trainees' comments on the training program seem to be taken seriously by the staff	0.50
		The guidelines clearly outline when to request input from a supervisor	0.49
	Task clarity	**The staff is clear about my duties and responsibilities** ^b^	0.56
		My workload in this job is fine^c^	0.51
		Senior medical staff is clear about what they expect of me during this term^b^	0.48
		**My program director prevents me from having to perform too many tasks irrelevant to my learning** ^c^	0.45
		There are clear clinical protocols in this post^b^	0.31
		There is clarity about to whom to report, in a variety of circumstances^b^	0.30
		I had an informative introductory program^b^	0.29

^a^ items selected for the abbreviated SPEED are presented in boldface

^b^ subtheme *communication*

^c^ subtheme *task demarcation*

### Item reduction and validation

Out of a total of 666 residents who were mailed the final draft instrument, 223 responded (33.5%). After deleting 15 respondents with incomplete responses, 208 completely scored final draft versions of the SPEED were available for analysis (S1 File). Cronbach’s α of item responses in the three domains were 0.87 for content, 0.89 for atmosphere, and 0.82 for organization, indicating high internal consistency. The correlation between mean item score per domain and the overall numerical score was 0.63, 0.67 and 0.65, respectively. Although this confirmed the high internal consistency of each domain, the correlation coefficients were below the predefined threshold of 0.85. Consequently, item reduction was performed using the internal consistency of the items within each domain and their representation of the entire domain in half of the database (the derivation dataset). In this derivation dataset, Cronbach’s α values for the content, atmosphere and organization domains of the final draft were 0.88, 0.90 and 0.82, respectively. The adjusted item-to-total domain score correlation coefficients of all items in the final draft SPEED as scored by the 104 respondents in the derivation dataset are presented in Table 1. For each subject theme, items were ranked by the strength of the adjusted correlation between item score and total domain score. As the content and atmosphere domains each contained five subject themes, we included from each theme the item with the highest item-to-total domain score correlation in the abbreviated instrument. The organization domain contained only three subject themes. To include 5 items from this organization domain into the abbreviated instrument, we selected one item from the subject theme with the lowest number of items, and two items from the other two subject themes (organization of supervision and task clarity). The two items from these subject themes were chosen to ensure representativeness of the subject theme in the final instrument (Table 1). Because the latter subject theme–task clarity–contained two subthemes (communication and task demarcation), we tried to ensure full coverage of this theme by selecting per subtheme the item with the highest item-to-total domain score correlation. As the item with the highest item-to-total domain score for task demarcation (“My workload in this job is fine”) had an insufficient item-to-total correlation in the validation dataset (<0.30), we substituted this item with the second highest item-to-total domain score correlation. This resulted in a satisfactory correlation in both the derivation and the validation dataset (.45 and .56 respectively). The internal consistency of the abbreviated SPEED as a whole was high (Cronbach’s α = 0.90). The internal consistencies of the three abbreviated scales in the derivation dataset were satisfactory to high, with Cronbach’s α values of 0.77, 0.80 and 0.67, respectively.

Validation of the abbreviated 15-item SPEED was performed on the responses of the 104 respondents in the validation dataset. The item-to-total domain score correlations for each of the 15 items in the abbreviated instrument in this validation dataset are presented in Table 2. The internal consistencies of the SPEED – 0.86 for the SPEED as a whole and 0.71, 0.75 and 0.67, respectively, for the 3 scales–were comparable to those found in the derivation dataset. The correlations between total domain scores of the draft and abbreviated instruments in the validation dataset were high (0.87, 0.94 and 0.89, respectively), and highly significant (p<0.001). The variance in each of the draft domain scores was largely explained by the respective items included in the final SPEED (85%, 89% and 80%, respectively). The differences between mean domain scores of the draft and abbreviated instruments (ranging from 0.02 to 0.07) were only small (Table 3).

**Table 2:** Items in the abbreviated version of the Scan of Postgraduate Educational Domains (SPEED), grouped per domain and subject theme, with their adjusted correlation coefficients to total domain score in the validation dataset.

	Item-to-total correlation
**Content** (Cronbach’s α 0.71)	
The feedback provided by my supervisor is focused on my strengths and weaknesses	0.52
In this rotation, evaluations are useful discussions about my performance	0.45
My supervisors are all in their own way positive role models	0.52
The level of autonomy given to me is appropriate to my level of training	0.35
The training in this post prepares me for my future career	0.53
**Atmosphere** (Cronbach’s α 0.75)	
The supervisors are approachable and helpful	0.61
Supervisors, nursing staff, other allied health professionals and residents work together as a team here	0.50
There is (are) NO attending physician(s) who have a negative impact on the educational climate	0.49
My supervisor supports me in difficult situations (e.g. handover)	0.43
The supervisors are respectful towards residents	0.63
**Organization** (Cronbach’s α 0.67)	
Teaching and learning are emphasized in this department	0.58
Good clinical supervision is available at all times	0.30
The staff is clear about my duties and responsibilities	0.36
My program director reserves time to supervise/counsel me	0.47
My program director prevents me from having to perform too many tasks irrelevant to my learning	0.41

**Table 3:** Mean (SD) domain scores for the final draft and abbreviated versions of the Scan of Postgraduate Educational Environment Domains (SPEED).

	Final draft SPEED		Abbreviated SPEED	
	M	SD	M	SD
Content	1.85	0.35	1.83	0.43
Atmosphere	1.94	0.40	1.87	0.45
Organization	1.89	0.36	1.85	0.41

## Discussion

The aim of this study was to develop a concise valid instrument based on a solid theoretical framework, suitable for regular assessment of the PGME environment. For this purpose, we integrated a thorough theoretical basis with the content of existing instruments to assess the quality of the (postgraduate) medical educational environment. This approach resulted in an instrument of only 15 items that comprehensively covers the three key elements of the (postgraduate) medical educational environment: content, atmosphere and organization of education. We called this instrument the Scan of Postgraduate Educational Environment Domains (SPEED). Our study, which encompassed several development and validation stages, supports the face and content validity of the SPEED. Therefore, the SPEED is suitable for regular assessment of the PGME environment.

The fact that our study resulted in an instrument that is both concise and valid may be attributable to the integrated approach we applied. First, we founded the SPEED on a solid theoretical framework to ensure that it covers the key elements of good PGME quality. Second, we selected relevant content from previously developed instruments. Building on this previous work was not only efficient, but also considered justifiable because of the high quality of these existing instruments: they were developed carefully, published in peer-reviewed journals, the items fit into the theoretical framework that we adopted and were found to cover the educational environment comprehensively [3]. Third, to ensure complete coverage of each domain, we involved representatives of important stakeholder groups in the development process. This process encompassed a Delphi procedure and interviews that allowed unrestricted input from residents. Finally, during the item reduction stage, which we based on half of the dataset, we did not only take internal consistency into account, but–as recommended–we also paid heed to item content [27] by identifying subthemes within the domains and by selecting at least one item per predefined subtheme. In this way, we intended to ensure full representation of the underlying concepts and to avoid item redundancy on account of basing item reduction entirely on statistical criteria [27]. To ensure that this approach actually resulted in a sound instrument, we validated the SPEED using the other half of the dataset.

The outcomes of the validation process provided support for the quality of the abbreviated instrument. Its internal consistency was high and the item-to-total domain score correlations were adequate. In addition, the outcomes of the SPEED were strongly representative of the scores obtained with the full draft version, indicating high content validity.

Compared to the instruments to evaluate the quality of the PGME environments that have been published previously [6,19,20], the SPEED offers a number of potential advantages. First, the SPEED appears to be the first PGME environment instrument founded on a solid theoretical framework [3]. Leading authors in the field of medical education have emphasized the value and importance of explicitly formulating and applying a theoretical framework for achieving best practice and moving the educational research field forward [11–13]. By developing the SPEED according to the recommendations of these experts, we intended to develop a scientifically sound instrument of high quality. Second, we built upon earlier work by using existing instruments as a basis for the SPEED. The fact that we integrated their contents–which vary in concepts measured–with a solid theory, may add to the field and help to converge the lines of educational environment research towards a joint language and frame of reference. According to the framework on which the SPEED is based, an excellent PGME program ensures optimal content, good atmosphere, as well as appropriate organization of education. Several contemporary studies of the clinical educational environment [29–33] report comparable key elements of the clinical learning setting, supporting the appropriateness of the theoretical framework we used. Considering the importance of educational environment quality for both learning effectiveness and trainee wellbeing [2,4], supervisors should be made aware of all essential conditions and take care of meeting these conditions. Therefore, it is vital not to restrict PGME evaluation to the contents of the program, but to also include atmosphere and organization. The SPEED may serve as an appropriate tool to assess whether PGME programmes are up to standard on each of these domains.

Compared to earlier instruments assessing educational environment quality, a third and final advantage of the SPEED is its conciseness, with only 15 items to be scored instead of up to 50 [19,20]. The main reason why researchers may prefer a long questionnaire is to ensure comprehensive coverage of the concept to be assessed. The drawback of an instrument with long lists of items to be answered, however, is its adverse effect on response rates. Studies from within and outside the medical field showed that response rates were substantially higher when questionnaires were shorter [21,22,34]. Although we acknowledge the complexity of the PGME environment [3,35], the current study shows that it can be evaluated comprehensively with an instrument of only 15 items.

### Strengths and limitations

The main strength of our study is the thorough and innovative development process of this PGME environment instrument, building on a sound theoretical framework and previous research, involving stakeholders, and basing the item reduction process on recommendations from experts in the field. The main limitation of our study is the relatively low response rate during the validation phase. We would like to emphasize that this was the response to a 43-item questionnaire with item redundancy and that response rates may be higher when using the final, abbreviated SPEED. The sample size was large enough, however, to perform the validation analyses, even those in which only half of the dataset was used. Besides, we did not restrict item reduction to a statistical procedure by basing it entirely on the analyses, but we also took item content into account. In addition, to prevent item reduction on the basis of idiosyncratic characteristics of the data, we validated our item selection using a dataset different from the development dataset. The fact that the outcomes of the SPEED are representative of those obtained with the full draft version is promising and supports our development process as sound and the SPEED as a valid tool to obtain an apt and encompassing picture of the quality of the PGME environment. Using the contents of previously developed environment instruments may pose a second limitation to our study. However, despite the fact that existing instruments in our field lacked theoretical frameworks, we consider their contents as a useful starting point for our work. The existing instruments were developed conscientiously through profound qualitative research methods, in various cases even using more than one (qualitative) research approach, such as literature review, grounded theory which often involved stakeholders through Delphi panels and/or focus groups, and/or using existing instruments from within or outside the medical educational field. As a result, the instruments appear to contain rich materials, which are considered valid to practice by important stakeholders. Besides, the fact that they were all published in peer-reviewed journals represents an acknowledgment of the quality of the instruments. Also, their wide coverage of environment aspects makes them suitable as an adequate basis for judging the relevance of items for the new instrument to be developed. A third possible limitation is that the final SPEED may provide less diagnostic information than a long questionnaire due to its brevity. As our study shows, however, the items that we selected for inclusion in the final SPEED explained a high percentage of the variance and therefore, it is suitable to indicate problems in (certain domains of) the educational environment. In case of such problems, longer questionnaires might be administered or other kinds of evaluation procedures started, for instance interviews may be held, to explore the problematic aspects in-depth in order to resolve them.

Future research might focus on further validation of the SPEED. As explained by Stanton et al. [27] item completion is dependent on the surrounding items in the questionnaire. Therefore, cross-validating the SPEED in a new data collection effort without the discarded items (i.e. administering the reduced length SPEED rather than the 43-item draft instrument) is important to verify its psychometric properties. Construct validity in terms of convergent and divergent validity could be further studied by investigating relationships of SPEED scales scores with those of other scales that can be expected to be more or less related with them. Furthermore, to find out how many respondents are needed for reliable outcomes, generalizability research is necessary.

### Conclusions

We succeeded in our aim to develop a concise instrument of only 15 items to assess the quality of the educational environment in PGME, which seems feasible for regular application. In the development process, we followed the suggestions of experts to use a guiding theoretical framework and build upon previous work [11–13]. Our first findings indicate that the SPEED is suitable to screen the quality of the educational environment in PGME programs on a regular basis. Further validation of the SPEED may not only support the guiding theoretical framework as sound, but may also help to converge the lines of educational environment research towards a joint language and frame of reference.

## Supporting Information

S1 File this file is an SPSS data file containing the anomymized data set of the 208 completely scored final draft versions of the SPEED which were available for item reduction and validation (SAV)
